# Design and Analysis of Circular Polarized Two-Port MIMO Antennas with Various Antenna Element Orientations

**DOI:** 10.3390/mi14020380

**Published:** 2023-02-03

**Authors:** Fatma Taher, Hussam Al Hamadi, Mohammed S. Alzaidi, Hesham Alhumyani, Dalia H. Elkamchouchi, Yasser H. Elkamshoushy, Mohammad T. Haweel, Mohamed Fathy Abo Sree, Sara Yehia Abdel Fatah

**Affiliations:** 1 College of Technological Innovation, Zayed University, Dubai 19282, United Arab Emirates; 2 College of Engineering and IT, University of Dubai, Dubai 14143, United Arab Emirates; 3 Department of Electrical Engineering, College of Engineering, Taif University, P.O. Box 11099, Taif 21944, Saudi Arabia; 4 Department of Computer Engineering, College of Computers and Information Technology, Taif University, P.O. Box 11099, Taif 21944, Saudi Arabia; 5 Department of Information Technology, College of Computer and Information Sciences, Princess Nourah bint Abdulrahman University, P.O. Box 84428, Riyadh 11671, Saudi Arabia; 6 Electrical Engineering Department, Faculty of Engineering, Pharos University, Alexandria 21311, Egypt; 7 Electrical Engineering Department, Shaqra University, Riyadh 17454, Saudi Arabia; 8 Electronics and Communication Engineering Department, Al-Madinah Higher Institute for Engineering and Technology, Giza 12947, Egypt; 9 Department of Electronics and Communications Engineering, Arab Academy for Science, Technology and Maritime Transport, Cairo 11865, Egypt; 10 Deparment of Electronics and Communication, Higher Institute of Engineering and Technology, EI-Tagammoe EI-Khames, Cairo 11835, Egypt; 11 Department of Electrical Engineering, Faculty of Engineering, Egyptian Chinese University, Cairo 11771, Egypt

**Keywords:** CP Antenna, LHCP, RHCP, MIMO, 28GHz

## Abstract

This article presents the circularly polarized antenna operating over 28 GHz mm-wave applications. The suggested antenna has compact size, simple geometry, wideband, high gain, and offers circular polarization. Afterward, two-port MIMO antenna are designed to get Left Hand Circular Polarization (LHCP) and Right-Hand Circular Polarization (RHCP). Four different cases are adopted to construct two-port MIMO antenna of suggested antenna. In case 1, both of the elements are placed parallel to each other; in the second case, the element is parallel but the radiating patch of second antenna element are rotated by 180°. In the third case, the second antenna element is placed orthogonally to the first antenna element. In the final case, the antenna is parallel but placed in the opposite end of substrate material. The S-parameters, axial ratio bandwidth (ARBW) gain, and radiation efficiency are studied and compared in all these cases. The two MIMO systems of all cases are designed by using Roger RT/Duroid 6002 with thickness of 0.79 mm. The overall size of two-port MIMO antennas is 20.5 mm × 12 mm × 0.79 mm. The MIMO configuration of the suggested CP antenna offers wideband, low mutual coupling, wide ARBW, high gain, and high radiation efficiency. The hardware prototype of all cases is fabricated to verify the predicated results. Moreover, the comparison of suggested two-port MIMO antenna is also performed with already published work, which show the quality of suggested work in terms of various performance parameters over them.

## 1. Introduction

The rapid advancement in wireless communication technologies is observed in the current era. The number of users is increasing day by day, along with high data rate demand [1]. For this purpose, researchers are working on solving the problem to facilitate the ocean of users with the best services. To do so, the requirement is revised to design a wireless communication system, which directly effects and changes the requirements of antenna designing [2]. To overcome the aforementioned problem, compact, wideband, high gain, simplified geometry antennas are in demand [3]. Currently, millimeter wave spectrum is utilized to obtain the high data rate transmission, as well as circularly polarized (CP) antennas, which are a good option for stable communication due to their resilience to multipath interference and ability to provide polarization mismatching between transmitting and receiving antenna [4,5]. Due to this application of CP antenna, the CP antenna are highly used in satellite communication, RFID, and future 5G base station and mobile communications [6]. In literature, numerous CP antenna were designed for lower bands (GPS, ISM, WLAM, and 5Gsub-6GHz) [7,8,9,10] and higher bands (mm-wave spectrum) [11,12,13,14,15,16,17,18,19,20,21]. The CP antennas are also made capable of operating at left-hand side, called Left Hand Circularly Polarization (LHCP), and right-hand side, called Right Hand Circularly Polarization (RHCP).

CP antenna offering simultaneous LHCP and RHCP for GPS communication is reported in [7]. The antenna operates at 1.56 GHz and offers an axial ratio of <1.35. The overall dimension of the antenna is 100 mm × 100 mm and has simple geometrical configuration. The two-port MIMO antenna reported in [8] contains overall dimensions of 22.5 mm × 50 mm. The reported work operates at 5.6 GHz and offers an axial ratio over 5.37–5.72 GHz. A low-profile CP antenna having simple geometry and loaded with meta-surface is given in [9]. The antenna offers bandwidth of 4.28–6.37 GHz and axial ratio bandwidth of 5.18–6.29 GHz. In [10], the reconfigurable CP antenna is reported for three bands of 2.5, 3.3, and 3.8 GHz. The antenna offers LHCP and RHCP ranging from 2.47–2.56 GHz, 3.2–3.42 GHz, and 3.74–3.83 GHz.

In literature, a number of CP antenna are designed for millimeter wave applications as well. In [11], a frequency reconfigurable patch antenna is given for 28 GHz 5G applications. The antenna has simple geometry and a compact size of 19 mm × 17 mm but offers narrow axial bandwidth of 1.7 GHz and 1.8 GHz at resonance frequencies of 28 GHz and 38 GHz, respectively. A reflect array antenna for satellite communication is reported in [12]. Although the antenna offers wide axial ratio bandwidth of 2.35 GHz, it has a large dimension of 180 mm × 180 mm, as well as complex geometry. Substrate integrated waveguide (SIW) antenna for millimeter wave applications offering LHCP and RHCP is given in [13]. The antenna has simple geometry but a large size of 70 mm × 63.5 mm × 2.2 mm and a narrow ARBW of 0.6 GHz. 

In [14], a series slot-fed MIMO antenna array with overall size of 28.7 mm × 27 mm × 2.5 mm and offering high gain of 11.6 dBi is given. The antenna offers an axial ratio bandwidth of 27.3–29.65 GHz. Although the antenna has compact size, high gain, and wideband axial ratio, the setback is complex geometry. A meta-surface-based antenna for 28 GHz applications is reported in [15]. The antenna has a compact size of 20.4 × 20.4 × 0.5 and has simplified geometry. The antenna has a high gain of 11 dBi and offers an axial ratio bandwidth of 3.4 GHz. The antenna mechanism is complex due to the loading of meta surface. Aperture couple antenna array for satellite communication is reported in [16]. The antenna offers a wide axial ratio and high peak gain but has large size and complex geometry. In [17], CPW-fed dual band antenna offering LHCP and RCHP is given. The antenna has a compact size of 10 mm × 10 mm × 0.51 mm but a low gain of 0.51 dBi. Another compact CP antenna is reported in [18]. The antenna has an overall size of 20 mm × 20 mm and offers 28.5–32 GHz axial ratio bandwidth. The setback of this reported work is complex geometry and low gain of antenna. 

A wideband antenna operating at 28 GHz with a bandwidth of 24.2–30.4 GHz is reported in [19]. The antenna is 18.1% axial ratio and has a peak gain of 8.5 dBi. Another work in [20] offers LHCP and RHCP but has complex geometry. The antenna has offers 14.5 dBi and 40% axial ratio bandwidth over K and Ka-band applications. In [22], a novel antenna offering broadband, reconfiguration, and circular polarization is reported. The rotation of the E-shaped patch and its array is utilized to obtain reconfiguration. The reported work offers LHCP and RHCP but has a large geometry of 44.1 mm × 78 mm. A millimeter wave antenna operating at 28 GHz is reported in [23] for circular polarization applications. The antenna offers a maximum gain of 21.9 dBi and axial ratio bandwidth of 3 GHz. Although the antenna has high gain and offers wide axial ratio bandwidth, demerit is complex geometry, due to travelling wave feeding approach.

From the above discussion and brief literature review, it is clear that there are still research gaps/problems with the design antenna having compact size, simple geometry, low-profile and offers wideband, high gain, and wide axial ratio bandwidth for 5G applications. Therefore, in this paper, an antenna is designed and investigated to meet the requirements of current and future 5G devices operating over a millimeter wave band spectrum of 28 GHz. In this article, a simplified structure, compacted size, low-profile, wideband, high gain, and high radiation efficiency antenna is proposed for millimeter wave applications. The antenna operates at 28 GHz band and is able to operate at LHCP and RHCP. The two-port antenna is designed to obtain the circular polarization in the left and right side. The MIMO antenna element is placed in various places at substrate to examine and study the polarization behavior of the MIMO antenna system. The antenna designing technique and effect of key parameters are given in the next section. In section III, the antenna MIMO configuration is explained by placing the patch element in various orientations. The proposed work is concluded in Section 4, along with references.

## 2. Design of Broadband CP Antenna

In this section, the circularly polarized antenna single element given in [24], is discussed, along with results. The reference antenna is designed on a commercially famous electromagnetic software tool known as HFSS.

### 2.1. Antenna Design Approach

**Figure 1:**
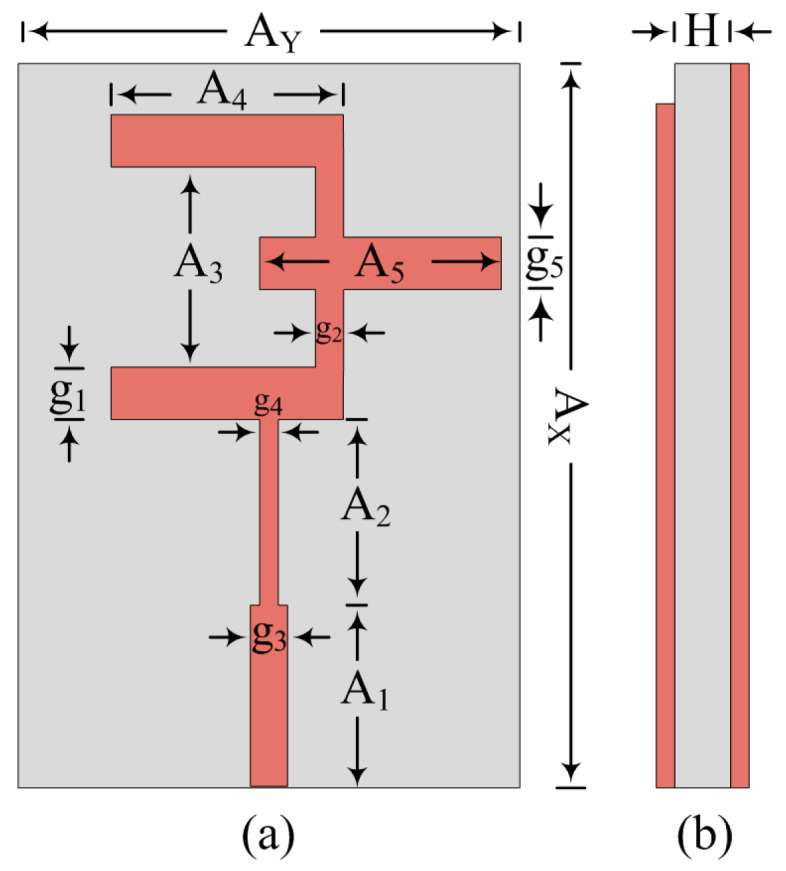
Antenna schematic (**a**) top-view (**b**) side-view.

**Figure 2:**
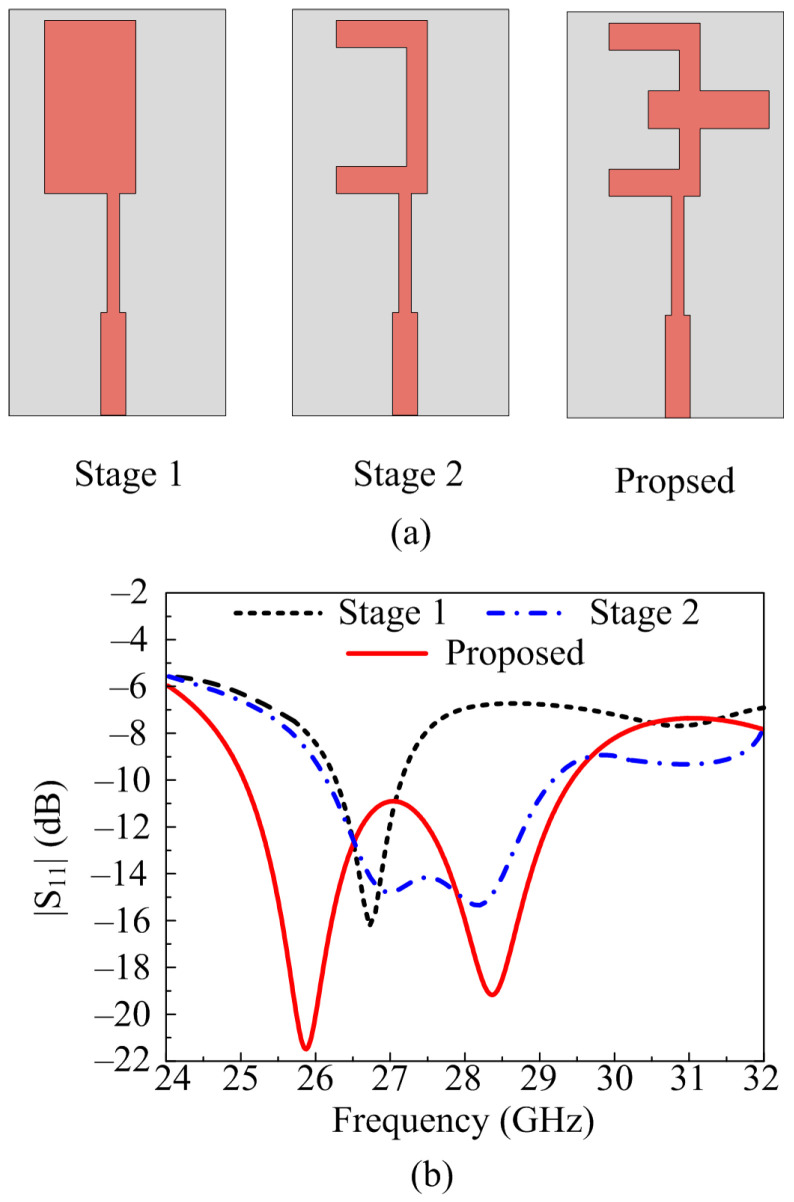
Antenna design steps (**a**) geometrical configuration (**b**) return loss.

The reference antenna is designed by implementing the two-stage feeding technique to obtain the miniaturized size, as well as impedance matching of 50 Ω. The design has the two-stage feeding feedline and the E-shaped patch. The antenna is planted over Roger RT/Duroid 6002, which has 2.94 and 0.0012 relative permittivity and loss tangent. The simplified geometry of the reference antenna is given in Figure 1. The offered scattering is depicted in (b) of Figure 2. It can be noticed that the antenna offer a wideband of 25.5–29.5 GHz. The optimized parameters of reference work is as follows:

A_x_ = 12; A_y_ = 8.5; A_1_ = 3.7; A_2_ = 2.3; A_3_ = 3; A_4_ = 3.5; A_5_ = 3.5; g_1_ = 1; g_2_ = 0.5; g_3_ = 0.6; g_4_ = 0.5; g_4_ = 0.5; H = 0.79. (All units are in mm).

The reference antenna’s final geometry is obtained after following three design stages. In the first stage of design, the rectangular patch antenna with two-stage feeding is designed for 28 GHz. The dimensions of the rectangular patch are obtained from the equation given in [25]. In the second stage, the square stub is etched from the rectangular patch antenna and the C-shaped patch is obtained. This design operates at a wideband of 26.5–29 GHz with a minimum return loss value of −14 dB. To improve the return loss and bandwidth, in the final stage, a rectangular stub is loaded in such a way to make the antenna patch of the E-shape. The final geometry operates at a wideband of 25.5–29.5 GHz with a minimum return loss of <−20 dB. The design stages of antenna and its impact on S-parameter is provided in Figure 2.

### 2.2. Paraneteric Analysis

In this sub-section, the parametric analysis of two key parameters is given. The variation is noted in terms of return loss of antenna.

#### 2.2.1. Width of the Middle Stub (g_5_)

**Figure 3:**
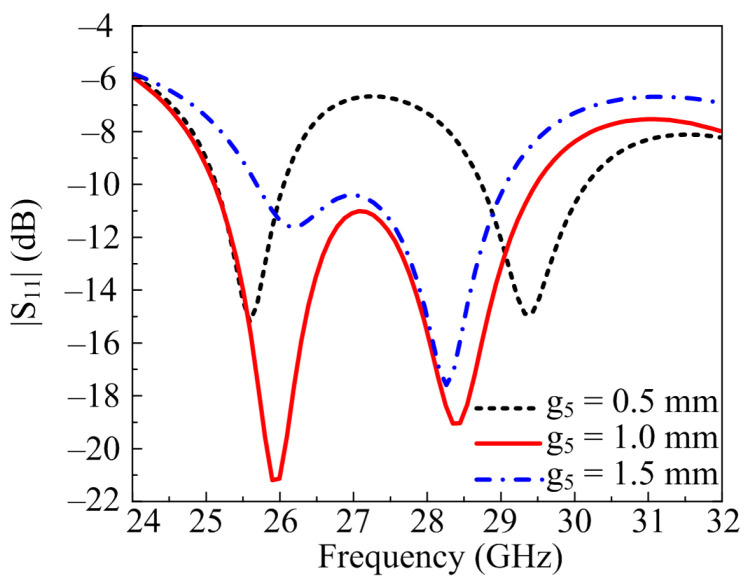
Parametric study of the stub width variation.

It is clear from previous discussions that the rectangular stub loaded to the antenna improves return loss and bandwidth. The width of the rectangular stub g_5_ is varied with different values to get the optimized value, as seen in Figure 3. At the optimal value of g_5_ = 1 mm, the antenna operates at wideband and offers a <−20 dB return loss. With the increase in value fixed on 1.5 mm, the wide band is compromised as well as the return loss. At this value, the antenna offers 26–29 GHz band with a <−17 dB return loss. On the other side, if the return loss is decreased to 0.5 mm, the wideband split into two small bands and operates at 25.25–25.8 GHz and 28.7–30.2 GHz with a return loss of <−15 dB.

#### 2.2.2. Width of the Extended Feedline (g_4_)

**Figure 4:**
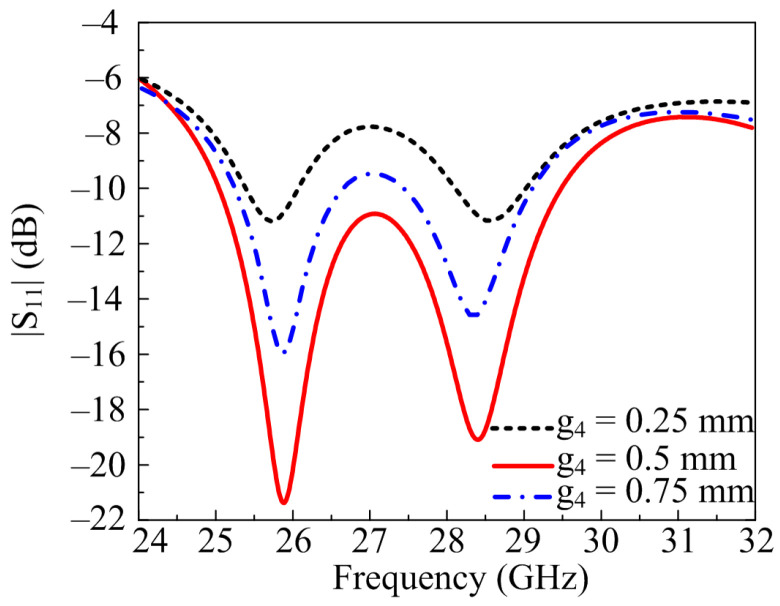
Parametric study of the extended feed width variation.

Another important parameter is the width of feedline, which is also cleared from design stages. At optimal value of g_4_ = 0.5 mm, antenna offers satisfactory results which are also discussed above. If the value of g_4_ is increased by 0.25 mm and fixed on 0.75 mm, the return loss is affected along with a slight effect on bandwidth. At g_4_ = 0.75 mm, the antenna offers a return loss <−15 dB. On the other side, if the value reduced from optimal value and was fixed on g_4_ = 0.25 mm, the return loss is compromised and reaches to <−11 dB, as given in Figure 4.

## 3. MIMO Antenna Configuration Analysis

### 3.1. Measurement Setup

**Figure 5:**
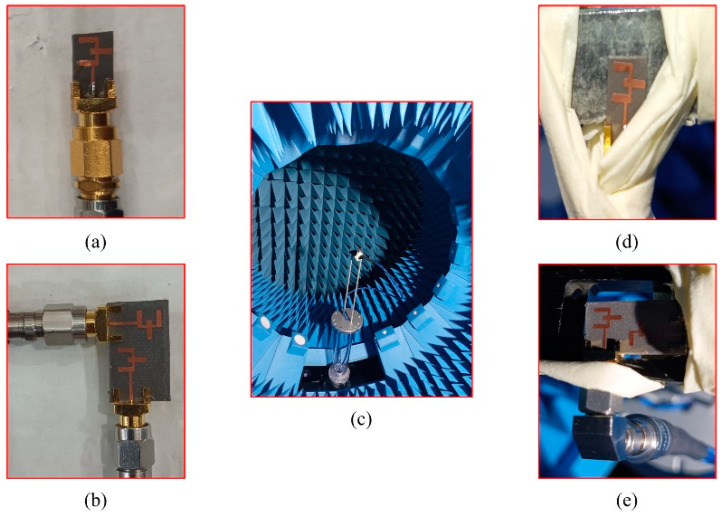
S-parameter measurements of (**a**) single-element (**b**) MIMO-antenna. (**c**) Far-field measurement setup, (**d**) single element and (**e**) MIMO element.

The sample fabricated prototype of the unit element, as well as the MIMO antennas, are utilized for measurement purposes. The performance is analyzed in terms of return loss and gain as well as axial ratio performance. Figure 5a,b shows the antenna prototype under s-parameters testing; the nearfield circular chamber for gain is shown in Figure 5c. The details about the circular chamber can be found in [26]. The close-up shots of the antenna under testing for gain measurements are depicted in Figure 5d,e.

### 3.2. Parallel Placement

**Figure 6:**
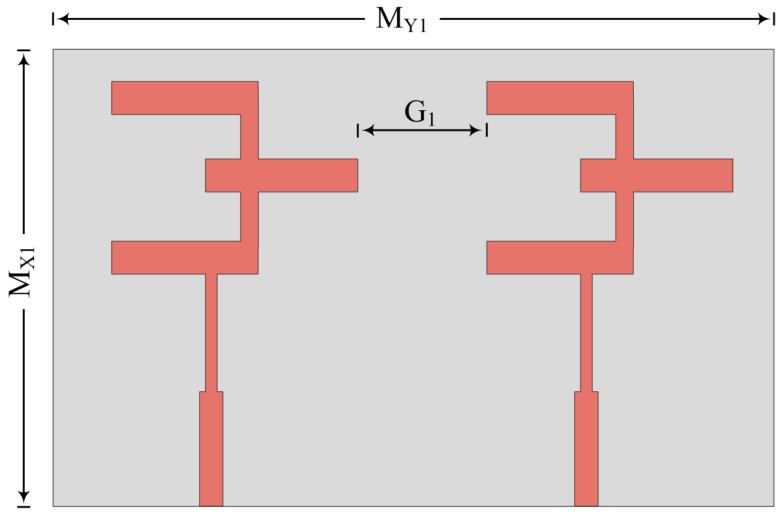
Schematic of MIMO antenna for parallel placement of elements.

In the first case, both antenna elements are placed parallel to each other with no changes in the radiating patch. The two-port MIMO antenna geometry along with the fabricated prototype is given in Figure 6. The two-port MIMO antenna has an overall size of M_X1_ × M_Y1_ = 20.5 mm × 12 mm. All of the parameters have the same value and same characteristics of substrate material, as discussed above. The space between two elements is fixed at G_1_ = 4.5 mm to nullify mutual coupling between them. To verify the suggested concept and methodology, the measurement of the prototype is performed and results are compared with predicated results obtained by EM tool HFSSv9 (High Frequency Structure Simulator).

**Figure 7:**
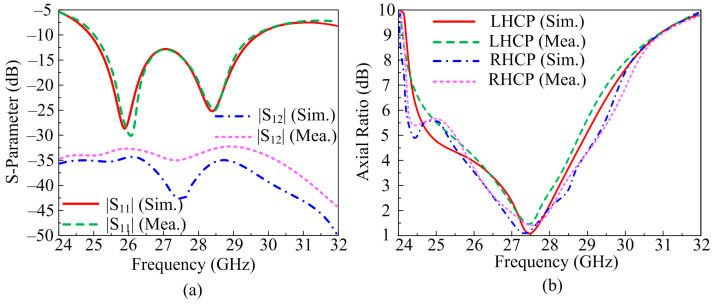
Comparison parameter of MIMO antenna with parallel element placement among (**a**) scattering parameters (**b**) axial ratio.

The scattering parameters of proposed two-port MIMO antenna operating over 28 GHz is given in Figure 7a. It can be verified from the depicted figure that the antenna offers a wide impedance band of 4 GHz ranging from 25.5–29.5 GHz. The two-port MIMO antenna system provides a return loss <−25 dB at resonance frequencies. The suggested design gives an excellent reading of transmission co-efficient. It is clear from the figure that the antenna offers S_12_ and S_21_ of <−35 dB at the operational band. The measured and predicated results did not have any significant differences which makes the suggested antenna a good applicant for future wideband MIMO antennas.

The AR (Axial Ratio) of the suggested antenna is depicted in Figure 7b. The proposed concept offers LHCP (Left Hand Circularly Polarization) and RHCP (Right Hand Circularly Polarization), as given. The predicated results, with the help of software, are verified by measuring hardware prototype results. It can be seen from the figure that the two-port MIMO antenna system offers a wide axial ratio bandwidth of 2.5 GHz of RHCP ranging from 26.3–28.7 GHz and 2.2 GHz of LHCP ranging from 26.7–28.5 GHz. The measured and simulated results have no major differences which make the suggested antenna a good candidate for future circularly polarized antenna.

**Figure 8:**
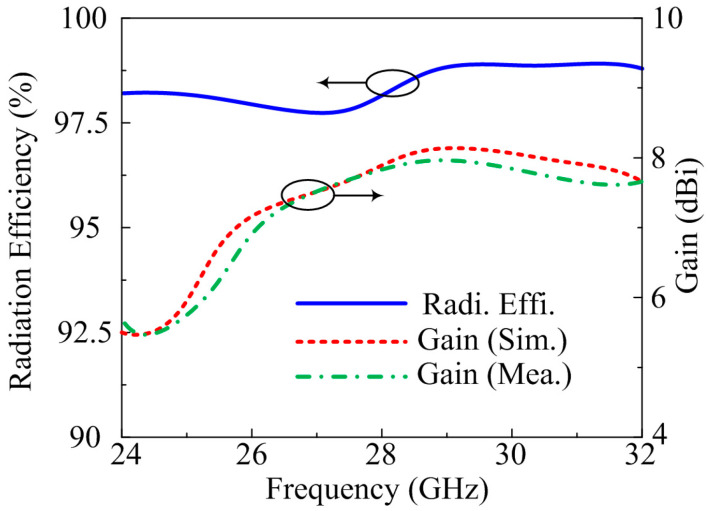
Comparison among gain and numerically calculated radiation efficiency of MIMO antenna of parallel placement of element.

The far-field parameters of the suggested antenna are also examined for further validation. The frequency versus gain plot, along with radiation efficiency, is given in Figure 8. The suggested design gives gain >7.75 dBi at operational bandwidth of 25.5–29.5 GHz with peak gain of 8.1 dBi at a resonance frequency of 28.5 GHz. Moreover, the radiation efficiency of >97.5% noted over operating bandwidth with a value of 98% is examined at 28.5 GHz. The predicated and tested result are quite similar, which implies that the proposed antenna is a good candidate for future high gain and high efficiency MIMO antenna devices.

### 3.3. Parallel Placement with Inverted Structure

**Figure 9:**
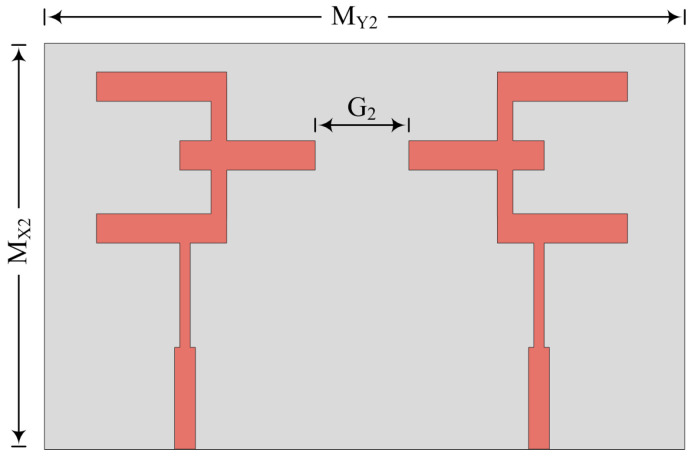
Schematic of MIMO antenna for inverted parallel placement of elements.

In this case, the MIMO antenna elements are parallel to each other, but the radiating patch is on the opposite side, as given in Figure 9. The suggested two-port MIMO antenna consists of an overall size of M_X2_ × M_Y2_ = 20.5 mm × 12 mm. The hardware prototype for this case is also fabricated to verify the predicated results. The space between two elements is fixed at G_2_ = 4 mm to nullify mutual coupling between them.

**Figure 10:**
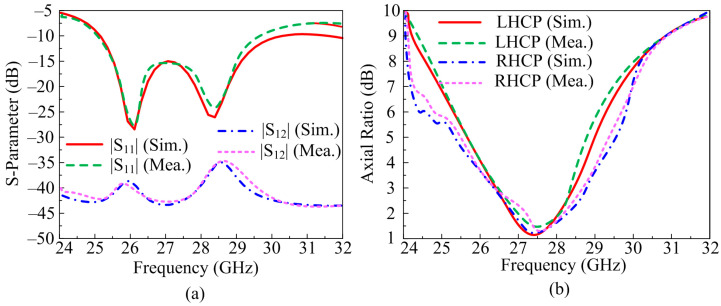
Comparison of results of MIMO antenna with parallel placement and inverted structure of element (**a**) scattering parameters (**b**) axial ratio.

The transmission and reflection co-efficient of the suggested two-port MIMO antenna system is depicted in Figure 10a. It can be seen from the figure that the antenna exhibits an over wide band of 4 GHz ranging from 25.4–29.4 GHz with a return loss value of <−25 dB. The antenna offers S12 and S21 of <−38 dB at operational bandwidth. These results infer that the antenna elements have no net effect on each other. The software predicated results and hardware prototype results have no major differences which makes the suggested design a good applicant for MIMO application on 28 GHz.

The AR of the suggested antenna is given in Figure 10b. It can be seen that the antenna offers a wide axial ratio bandwidth of 3 GHz for RHCP ranging from 26–29 GHz and 2.2 GHz for LHCP ranging from 26.3–28.5 GHz. Not much difference between software predicated results and prototype measured results is found, which makes the suggested antenna a a good candidate for future MIMO antenna devices in circular polarization application.

**Figure 11:**
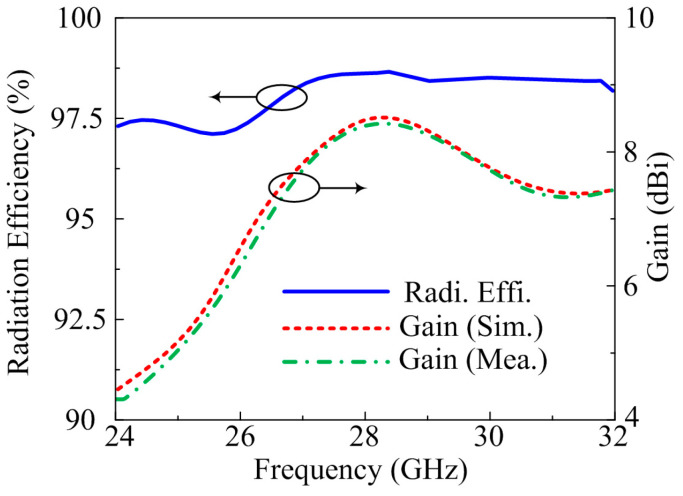
Comparison among gain and numerically calculated radiation efficiency of MIMO antenna with parallel and inverted structure element.

The frequency versus gain plot, as well as radiation efficiency of the suggested two-port MIMO antenna, is given in Figure 11. The antenna gives a gain > 7.5 dBi at operational bandwidth with peak value of 8.6 dBi at 28.5 GHz. From the figure, it is also noted that the suggested concept provides a radiation efficiency of >97.75% at operational bandwidth with a maximum value of 98.2% at 28.5 GHz. The predicated and tested results show many similarities which makes the suggested antenna a good candidate for future high gain and high efficiency devices.

### 3.4. Orthognal Placement

**Figure 12:**
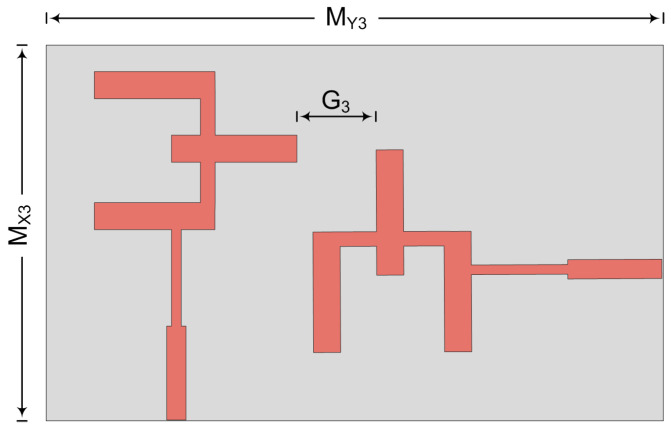
Schematic of MIMO antenna for orthogonal elements.

In this case, the two-port MIMO antenna is designed so that the second element is placed perpendicular to the first element, as given in Figure 12. The hardware prototype is also fabricated to verify the suggested concept. The suggested two-port MIMO system in this case has an overall size of M_X3_ × M_Y3_ = 20.5 mm × 12 mm. The space between the two elements is fixed at G_3_ = 3 mm to nullify mutual coupling between them.

**Figure 13:**
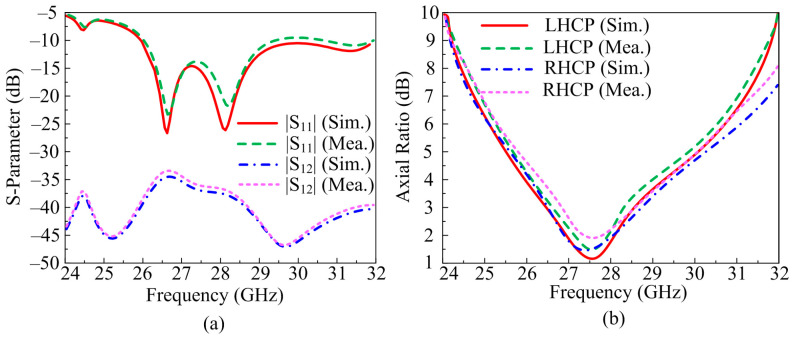
Comparison of results of MIMO antenna with orthogonal placement of element (**a**) scattering parameters (**b**) axial ratio.

The scattering parameter of the suggested two-port MIMO antenna is given in Figure 13a. It can be observed that the antenna offers a bandwidth of 3 GHz ranging from 26–29 GHz. At these frequencies, the antenna offers return loss <−25 dB. The most important parameter of the MIMO antenna is transmission co-efficient, where the effect of one element over other is examined. The antenna offers S_12_ and S_21_ < −35 dB, as given in the figure. The similarity between predicated and tested results are also observed, which implies that the antenna is a good applicant for MIMO antenna application operating over a mm-wave band. 

The AR of the proposed antenna is analyzed to show that the antenna offers both LHCP and RHCP. The results of ARBW are given in Figure 13b, which shows that the antenna gives AR of 2.75 GHz ranging from 26.25–29 GHz and AR of 3 GHz ranging from 26.5–28.5 GHz, for RHCP and LHCP, respectively. The figure shows that there is no major difference between predicated and prototype tested results, which leads the proposed MIMO system as the best applicant for CP antenna applications operating over 28 GHz.

**Figure 14:**
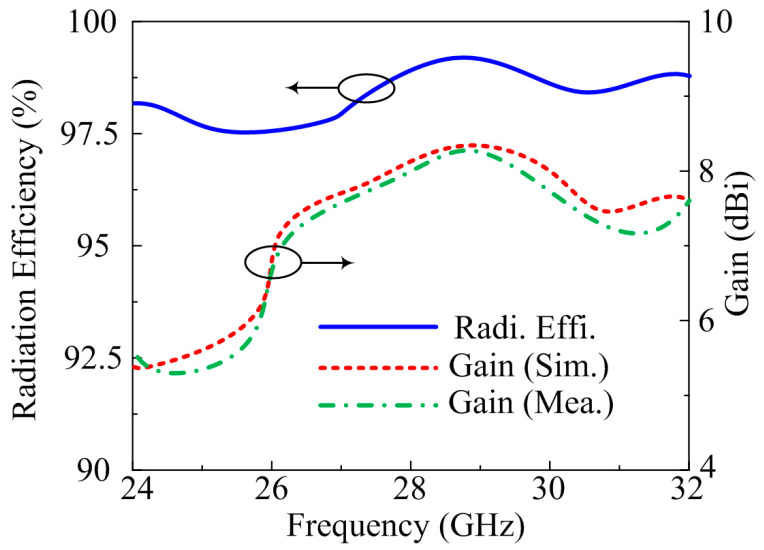
Comparison among gain and numerically calculated radiation efficiency of MIMO antenna with orthogonal placement of element.

Figure 14 depicts the frequency versus gain as well as radiation efficiency of the suggested two-port MIMO antenna. It can be noticed that the antenna offers gain > 7.6 dB at an operational band width of 26–29 GHz with a maximum gain value of 8.2 dBi at 28.5 GHz. The figure also expresses the radiation efficiency for the suggested antenna. The antenna offers the radiation efficiency > 97.5% at operational bandwidth with a maximum value of 98% at a resonance frequency of 28.5 GHz. The similarity between predicated and tested results is observed which makes the suggested design best for future high gain and high efficiency application antenna devices.

### 3.5. Inverted Structure Placement

**Figure 15:**
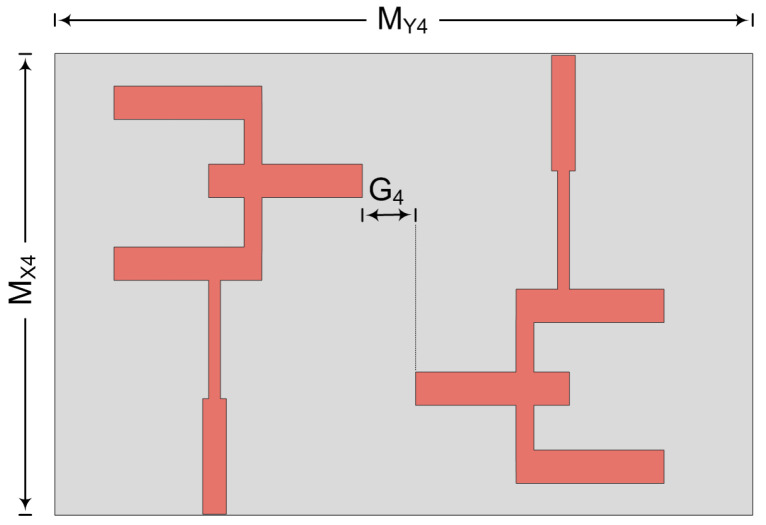
Schematic of MIMO antenna for opposite elements.

In this case, the two-port MIMO antenna is designed in such a way that the second element is placed parallel to the first element but on the opposite side of substrate, as given in Figure 15. The total dimension of the suggested two-port MIMO antenna is M_X4_ × M_Y4_ = 20.5 mm × 12 mm. The hardware prototype is also fabricated to verify the software generated results. The distance between the two elements in this case is G_4_ = 6 mm and fixed at this value to reduce mutual coupling between two elements.

**Figure 16:**
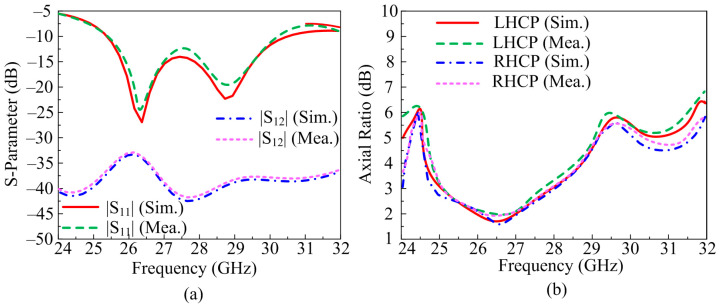
Comparison of MIMO antenna with inverted structure placement of element (**a**) scattering parameters (**b**) axial ratio.

Figure 16a depicts the transmission and reflection co-efficient of the suggested antenna. It can be noted from the figure that the suggested antenna two-port MIMO configuration operates at 4.5 GHz wideband ranging from 25.5–30 GHz. The antenna offers a return loss maximum of −20dB. The suggested antenna offers S_21_ and S_12_ of <−40 dB at operational bandwidth, which is much higher than the acceptable value. Moreover, the software predicated and hardware measured results show much similarity, which implies that the antenna is a good candidate for future wideband devices operating over a mm-wave spectrum.

The axial ratio bandwidth (ARBW) of the suggested antenna is given in Figure 16b. The antenna offers circular polarization of both left hand (LHCP) and right hand (RHCP). From the figure, it is clear that the antenna offers wide band AR of 3.1 GHz ranging from 25.2–28.3 GHz and 2.6 GHz ranging from 25.2–27.8 GHz for RHCP and LHCP, respectively. The figure also shows that there is no major difference between software predicated and prototype tested results, which makes the suggested antenna a good applicant for future mm-wave applications operating over circular polarization.

**Figure 17:**
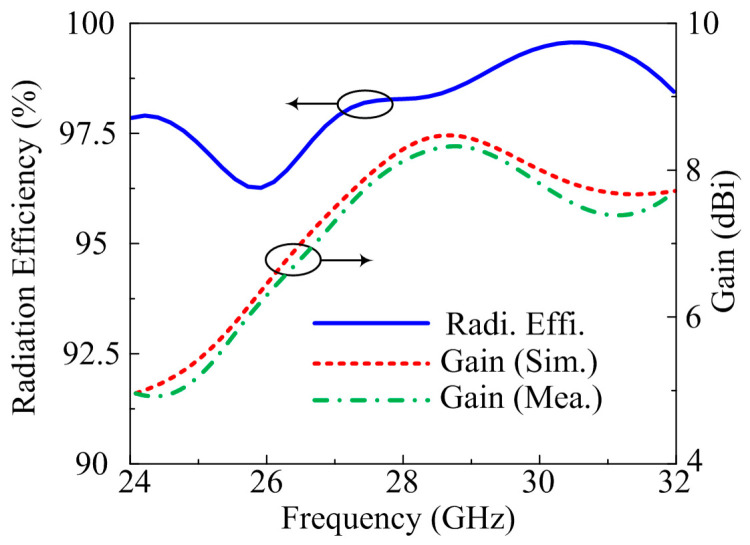
Comparison among gain and numerically calculated radiation efficiency of MIMO antenna with inverted structure placement of element.

Figure 17 shows the gain versus frequency as well as the radiation efficiency plot of the suggested antenna. It can be noted from the figure that the antenna offers gain > 7.8 dBi at operating bandwidth with a peak value of 8.75 dBi at 28.5 GHz. On the other hand, the antenna offers a radiation efficiency > 97.5% at operational band with a maximum value of 99% at 30 GHz. Moreover, the figure makes it clear that there is no major difference between software and hardware results, which makes the suggested design an excellent candidate for future high gain and high efficiency application.

### 3.6. Comparison among Various MIMO Configurations

The comparison between four designs differing on the bases of placement of the second antenna element of two-port MIMO antenna is given in Table 1. The results of two-port antennas differentiate on the bases of operational bandwidth, transmission co-efficient, ARBW, peak gain, and radiation bandwidth.

### 3.7. Comparison with State-of-the-Art

A number of works are presented in literature on operation over the 28 GHz spectrum and circular polarization. The dimension, operating frequency, bandwidth, gain, polarization type, ARBW, and antenna type are compared in the Table 2. It can be noted from the table that the proposed antenna either has compact size or operating over wideband and high gain, as compared to other listed work. Moreover, the suggested work also offers both LHCP and RHCP with wide ARBW. The mechanism used in this approach is simpler as compared to other work presented in literature.

## 4. Conclusions

Various cases of placing the second element of the two-port MIMO antenna are studied and verified by testing the hardware prototype in this paper. The antenna offers wideband and wide ARBW along with high gain. The two-port MIMO is constructed to get LHCP and RHCP. The second element of the two-port system is placed in various orientations to reference the first element to study the bandwidth, gain, and ARBW of the suggested mm-wave antenna. The suggested antenna MIMO systems offers wideband of 25.5–30 GHz with peak gain of 8.75 dBi. The antenna provides circular polarization with a wide bandwidth of 25.2–28.3 GHz, covering almost all bands of 28 GHz 5G applications. The reference antenna and its four cases of MIMO configuration are designed by using High Frequency Structure Simulator (HFSSv9). Moreover, the results in terms of bandwidth, mutual coupling, gain, and ARBW of all four cases are compared. Alternatively, the results of the suggested design are also compared with published work, which show that the suggested work offers wideband, high gain, wide ARBW, simple geometry compact size, offering both RHCP/LHCP and adoption of a simple design approach. The results, discussion, and comparison table show that the suggested two-port MIMO antenna is the best and most solid applicant for future mm-wave devices for CP applications.

## Figures and Tables

**Table 1:** Performance comparison of various MIMO configurations.

Antenna Design	Element Placement	Operational Bandwidth (GHz)	Transmission Co-Efficient (dB)	Maximum Axial Ratio Bandwidth (GHz)	Peak Gain (dBi)	Radiation Efficiency (%)
Antenna 1	Parallel placed	25.25–29.25	<−39	2.5	8.1	98
Antenna 2	Parallel placed with opposite direction	25.4–29.4	<−42	3	8.6	98.2
Antenna 3	Orthogonal placement	26–29	<−45	3	8.2	98
Antenna 4	Inverted placement	25.5–30	<−40	3.1	8.75	99

**Table 2:** Comparison of proposed study with state-of-the-art.

Ref.	Antenna Size mm^3^	Operating Frequency (GHz)	Bandwidth (GHz)	Gain (dBi)	Polarization Type	Axial Ratio Bandwidth (GHz)	Antenna Type and Mechanism
[11]	19 × 17 × 0.8	28 38	27–29.5 35–38.2	8 8.7	RHCP LHCP	27.3–29 35–38.2	Microstrip Patch antenna
[12]	180 × 180 × 1.67	19.7	17.2–22.7	-	RHCP LHCP	17–19	Reflect array Antenna
[13]	70 × 63.5 × 2.2	28	27–29.5	-	RHCP LHCP	27.7–28.3	SIW Antenna Array
[14]	28.7 × 27 × 2.5	28	26.9–30.7	11.86	CP	27.3–29.65	Series slot fed MIMO array
[15]	20.4 ×20.4 × 0.5	27.5	24.5–31	11	CP	25–29.3	Meta-surface-based antenna
[17]	10 × 10 × 0.51	32	28–39.2	12	CP LP	34–38	CPW fed Antenna with CRLH-MTM
[18]	20 × 20 × 0.254	30	26.5–34.7	9.5	RHCP LHCP	28.5–32	DRA array
[19]	10 × 10 × 15	28	24.2–30.4	8.5	CP	24.5–30	ME dipole Antenna
[22]	10 × 10 × 2.199	28	27.7–29.55	-	RHCP LHCP	27.7–29.25	SIW Array Antenna
This Work	20.5 × 12 × 0.79	28	25.5–30	8.75	RHCP LHCP	25.2–28.3	MIMO Configuration

## Data Availability

Not applicable.
